# A New Intervention for Implementation of Pharmacogenetics in Psychiatry: A Description of the PSY-PGx Clinical Study

**DOI:** 10.3390/ph17020151

**Published:** 2024-01-23

**Authors:** Teuntje A. D. Pelgrim, Alexandra Philipsen, Allan H. Young, Mario Juruena, Ester Jimenez, Eduard Vieta, Marin Jukić, Erik Van der Eycken, Urs Heilbronner, Ramona Moldovan, Martien J. H. Kas, Raj R. Jagesar, Markus M. Nöthen, Per Hoffmann, Noam Shomron, Laura L. Kilarski, Thérèse van Amelsvoort, Bea Campforts, The PSY-PGx Consortium, Roos van Westrhenen

**Affiliations:** 1 Department of Psychiatry, Parnassia Psychiatric Institute, 1062HN Amsterdam, The Netherlands; 2 Department of Psychiatry and Psychotherapy, University of Bonn, 53105 Bonn, Germany; 3 Department of Psychological Medicine, Institute of Psychiatry, Psychology and Neuroscience, King’s College London & South London and Maudsley NHS Foundation Trust, Bethlem Royal Hospital, Monks Orchard Road Beckenham, Kent BR3 3BX, UK; 4 Bipolar and Depressive Disorders Unit, Department of Psychiatry and Psychology, Hospital Clinic & Institute of Neurosciences (UBNeuro), IDIBAPS, CIBERSAM, ISCIII, University of Barcelona, 08036 Catalonia, Spain; 5 Department of Physiology, Faculty of Pharmacy, University of Belgrade, 11000 Belgrade, Serbia; 6 Department of Physiology & Pharmacology, Karolinska Institute, 171 77 Stockholm, Sweden; 7 Global Alliance of Mental Illness Advocacy Networks-Europe (GAMIAN-Europe), 1050 Brussels, Belgium; 8 Institute of Psychiatric Phenomics and Genomics (IPPG), LMU University Hospital, LMU Munich, 80336 Munich, Germany; 9 Department of Psychology, Babeş-Bolyai University, 400015 Cluj-Napoca, Romania; 10 Division of Evolution, Infection and Genomic Sciences, School of Biological Sciences, University of Manchester, Manchester M13 9PT, UK; 11 Manchester Center for Genomic Medicine, St. Mary’s Hospital, Manchester University NHS Foundation Trust, Manchester M13 9WL, UK; 12 Groningen Institute for Evolutionary Life Sciences, University of Groningen, 9700CC Groningen, The Netherlands; 13 Institute of Human Genetics, University Hospital of Bonn and University of Bonn, 53127 Bonn, Germany; 14 Faculty of Medicine, Tel Aviv University, Tel Aviv 69978, Israel; 15 Department of Psychiatry and Neuropsychology, Maastricht University, 6226NB Maastricht, The Netherlands; 16 St. John’s National Academy of Health Sciences, Bangalore 560034, India

**Keywords:** pharmacogenomics, mental disorders, personalized medicine, psychopharmacology, depressive disorders, anxiety disorders, psychotic disorders, schizophrenia, bipolar disorder, precision psychiatry

## Abstract

**(1) Background** Pharmacological treatment for psychiatric disorders has shown to only be effective in about one-third of patients, as it is associated with frequent treatment failure, often because of side effects, and a long process of trial-and-error pharmacotherapy until an effective and tolerable treatment is found. This notion emphasizes the urgency for a personalized medicine approach in psychiatry. **(2) Methods** This prospective patient- and rater-blinded, randomized, controlled study will investigate the effect of dose-adjustment of antidepressants *escitalopram* and *sertraline* or antipsychotics *risperidone* and *aripiprazole* according to the latest state-of-the-art international dosing recommendations for *CYP2C19* and *CYP2D6* metabolizer status in patients with mood, anxiety, and psychotic disorders. A total sample of N = 2500 will be recruited at nine sites in seven countries (expected drop-out rate of 30%). Patients will be randomized to a pharmacogenetic group or a dosing-as-usual group and treated over a 24-week period with four study visits. The primary outcome is personal recovery using the Recovery Assessment Scale as assessed by the patient (RAS-DS), with secondary outcomes including clinical effects (response or symptomatic remission), side effects, general well-being, digital phenotyping, and psychosocial functioning. **(3) Conclusions** This is, to our knowledge, the first international, multi-center, non-industry-sponsored randomized controlled trial (RCT) that may provide insights into the effectiveness and utility of implementing pharmacogenetic-guided treatment of psychiatric disorders, and as such, results will be incorporated in already available dosing guidelines.

## 1. Introduction

Psychiatric disorders are noncommunicable diseases that pose a major health challenge in Europe in the 21st Century [1,2]. In 2019, it was estimated that worldwide 970 million people were living with a psychiatric disorder [3], a number that has only risen since the COVID-19 pandemic and is expected to rise even more over time [1,3,4]. The economic burden of adults with major depressive disorder in the United States alone was estimated at 210.5 USD in 2010 [4]. As such, the growing global economic burden of unmet psychiatric health needs is putting a large strain on healthcare budgets. Our fundamental understanding of etiopathogenic mechanisms underlying psychiatric disorders, in particular in the field of genomics, has greatly advanced in recent years. For many years, however, the development of new drugs with fundamentally new therapeutic targets has unfortunately stagnated, leaving existing drugs as the only treatment alternatives to psychological interventions [5]. However, the use of these drugs is associated with frequent treatment failure, often due to side effects, and many patients are left with a long and arduous process of trial-and-error pharmacotherapy until they receive effective and tolerable treatment [6]. Pharmacological treatment for psychiatric disorders has shown to be only effective in about one-third of patients, resulting in a considerable number of therapy-resistant patients [5,6,7], an increased risk for comorbid diseases, and a reduced life expectancy of up to 20 years [3]. Moreover, the efficacy of effective treatments is limited by low compliance, often due to adverse effects [8]. These adverse effects are, in part, driven by the heterogeneity in genes encoding drug-metabolising enzymes [9]. Growing evidence suggests that genotyping may help to identify which patients benefit from what treatments, using so-called pharmacogenetics to personalize medicine, which can also be used in psychiatry [5,7,10,11]. These advances have not yet reached clinical practice on a larger scale [12,13].

By identifying genetic variations that influence the effectiveness and adverse effects of medications, more informed decisions about treatment can be made, leading to more effective treatment. Although pharmacogenetic dosing guidelines currently exist for over 100 medications, amongst others for antidepressants and antipsychotics [14,15,16], the clinical evidence of pharmacogenetic testing in psychiatry is still debated even though recent publications show clinical benefits [17,18] by reducing adverse drug reactions and maximizing the intended use of medications [19,20,21,22,23]. Previous prospective randomized controlled trials have demonstrated the positive effects of pharmacogenetics for antidepressants on clinical symptoms (recently meta-analyzed by [17]). A recent study showed that pharmacogenetic-guided antidepressant use in the treatment of major depressive disorder resulted in considerable population health benefits while substantially reducing healthcare costs, suggesting that implementing pharmacogenetic-guided treatment in psychiatry may have large healthcare and economic benefits [24,25]. However, the implementation of pharmacogenetics in psychiatry is still hampered, partly because previous prospective clinical trials were based on mostly small samples, which were (at least in part) industry-sponsored, all used commercial pharmacogenetic combinatorial panels and used so-called ‘black-box’ algorithms, in which the inner workings of the algorithm is unknown to users [22,23,26,27,28,29,30,31,32,33,34,35,36,37,38]. Additionally, the studies that were subject to the meta-analysis by Brown and colleagues in 2022 [17] were not very comparable in terms of drugs that were studied and time intervals, warranting the need for a coherent protocol, such as the work presented here. Most pharmacogenetic studies in psychiatry focus solely on participants with depressive disorders [17], overlooking the clinical utility of pharmacogenetics for patients with other psychiatric conditions [39]. Only one prospective trial included anxiety disorder as a comorbid disorder in addition to depressive disorder [32]. As there are pharmacogenetic guidelines available not only for antidepressants [40] but also for antipsychotics [41], there are other disorders of interest to include in pharmacogenetic clinical trials. Few studies have investigated the potential of pharmacogenetics in treating patients with psychotic disorders in prospective randomized control settings [20,42], and also patients who are diagnosed with anxiety disorders that are commonly treated with antidepressants could benefit, as was shown by Greden and colleagues [32]. A recent retrospective study found that pre-emptive genotyping may be valuable for individualizing antipsychotic dosing and treatment [43]. Thus, to address the main obstacle impeding the implementation of clinical guidelines of pharmacogenetics in psychiatry, larger prospective evidence on clinical outcomes is needed [44].

Several genes involved in psychopharmaceutical metabolism have been identified; specifically, the cytochrome P450 drug-metabolizing enzymes have been found to contribute to interindividual differences in antidepressant and antipsychotic metabolism [45]. Individuals can be classified into phenotypic subgroups based on inherited alleles that are associated with different classifications of drug metabolism. These groups include normal metabolizers (NMs), intermediate metabolizers (IMs), poor metabolizers (PMs), and ultra-rapid metabolizers (UMs). Most antidepressants and antipsychotics are metabolized by cytochrome P450 enzymes, and especially *CYP2C19* and *CYP2D6* have been shown to significantly influence psychopharmaceutic drug levels and, therefore, seem best fitted for clinical use in psychiatry [46,47,48]. The *CYP2C19* and *CYP2D6* genes are highly polymorphic with multiple allelic variants associated with altered enzymatic capacity, and their genotype status may predict treatment response in certain individuals [49]. The distribution of CYP allele variants differs considerably between populations [50]. However, more non-industry-sponsored research, also including non-mood disorder patients, is needed to determine whether this translates to improvement in clinical outcomes by genotype-guided treatment selection [21]. Therefore, we hereby present the protocol of the PSY-PGx Clinical Study, which is an international, non-industry-sponsored, prospective pharmacogenetic clinical study conducted at nine clinical sites in seven countries. Here, we aim to investigate the effect of pharmacogenetic-informed dose adjustment of the antidepressants escitalopram and sertraline or the antipsychotics risperidone and aripiprazole according to the latest state-of-the-art international dosing recommendations for *CYP2C19* and *CYP2D6* metabolizer status in patients with mood-, anxiety- or psychotic disorder. 

### Objectives

The main aim of the current study is to compare outcomes of individualized medication dosing based on pharmacogenetics with outcomes following dosing prescription as usual in patients diagnosed with psychotic disorders, depressive disorders, and anxiety disorders. To that end, we will assess patient recovery, as reported by patients themselves (Recovery Assessment Scale: RAS-DS), in response to psychopharmacological treatment from baseline to the end of week 24 of the study. The secondary objective is to evaluate the impact of pharmacogenetic testing on clinical response, symptomatic remission, side effects, general well-being, and psychosocial functioning. Further, patients will be phenotyped to identify other factors besides pharmacogenetics that may influence individual medication response, including passive monitoring of behavioral aspects by means of a mobile phone application. Finally, a further aim is to investigate other genetic factors related to medication response and pathophysiology, including genetic variants related to drug absorption, distribution, metabolism, and elimination. 

## 2. Experimental Design

### 2.1. Study Design

This international, multi-center study applies a prospective, 24-week randomized, patient- and rater-blinded, two-arm, parallel-group controlled, non-commercial trial of pharmacogenetic-informed pharmacotherapy. The basis for this trial is a non-commercial outpatient clinic on pharmacogenetics that was set up in Amsterdam by the Chief Principal Investigator in 2017. In this clinical trial, psychiatric patients with a diagnosis of anxiety-, mood- or psychotic disorders will be randomized in a 1:1 ratio to either the intervention (dosing based on pharmacogenetics) or a control condition (dosing as usual; DAU). The study is expected to recruit participants over a 2.5-year period (starting in 2023). 

All procedures contributing to this work comply with the ethical standards of the relevant national and institutional committees on human experimentation and are in line with the Helsinki Declaration of 1975 (revised in 2008). All procedures will be approved by the local ethics committees in each of the participating countries prior to any study-related activities, using either local ethics procedures or via the Clinical Trials Information System (CTIS). The study was registered at ClinicalTrials.gov (accessed on 17 January 2024, Identifier: NCT05656469).

### 2.2. Recruitment and Participants 

Written informed consent to participate in this study will be obtained from each participant prior to any study-related activities. All participants aged between 16 and years 70 years will be recruited from referrals of inpatient and outpatient facilities to the nine participating centers: Parnassia Groep BV (PGB), Farmaceutski Fakultet Univerzitetau Beogradu (FFUB), University Hospital Bonn (UKB), Maastricht University (MU), LMU University Hospital Munich (LMUM), King’s College London (KCL), Fundació Clínic per a la Recerca Biomèdica (FCRB), Universitatea Babeş-Bolyai (UBB), and SUNY Upstate Medical School, Syracuse, NY, USA (SUNY). All participants will have a primary diagnosis of one or more of the following diagnoses. (1) Mood disorder (major depressive disorder or bipolar disorder (currently depressive episode). (2) Anxiety disorder (e.g., panic disorder, generalized anxiety disorder). (3) Psychotic disorder (schizophrenia or schizoaffective disorder). All diagnoses will be confirmed using psychiatric evaluation in agreement with DSM-5 criteria by means of the Mini-International Neuropsychiatric Interview (MINI) [51]. 

### 2.3. Inclusion and Exclusion Criteria

The inclusion criteria for participants are: (a) diagnosed with a depressive episode (major depressive disorder or bipolar disorder; currently depressive episode), and/or an anxiety disorder (e.g., panic disorder, social phobia, specific phobia, agoraphobia, generalized anxiety disorder) and/or a psychotic disorder (schizophrenia and schizoaffective disorder) of at least moderate severity as assessed using the Structured Interview Guide for the Hamilton Depression Scale (SIGH-D; score of 14 or higher), the Structured Interview Guide for the Hamilton Anxiety Scale (SIGH-A; score of 18 or higher), or the Positive and Negative Syndrome Scale (PANSS; score of 75 or higher), respectively; (b) previous inadequate response to at least one psychotropic treatment (defined as insufficient efficacy of a psychotropic treatment when dosed high enough and maintained long enough, or discontinuation of a psychotropic treatment due to adverse events or intolerability); (c) about to switch medication to sertraline or escitalopram (for patients with mood or anxiety disorders), or to aripiprazole or risperidone (for patients with psychotic disorders), or have switched within the last 2 weeks prior to first contact with an investigator; (d) receiving inpatient or outpatient psychiatric treatment; (e) understanding requirements of the study and providing written informed consent; (f) possessing a mobile phone (Android or iOS operation system) for passive behavioral monitoring.

The exclusion criteria include (a) prior pharmacogenetic testing; (b) no prior use of psychotropic medication; (c) severe somatic comorbidities as reported in participant’s medical history or based on clinical chemistry and electrocardiogram (ECG) (e.g., liver disease: alanine-aminotransferase (ALAT) > 70 u/L; renal disease: estimated glomerular filtration rate (eGFR) < 60 mL/min/1.73 m^2^; diabetes: blood glucose > 11.1 mmol/L or two times fasting glucose > 7.0 mmol/L; cardiac disease: prolonged QT-interval) (d) alcohol and substance abuse or dependence (except nicotine), (e) polypharmacy defined as routine use of 5 or more medications (including over-the-counter, prescription and/or traditional and complementary medicines), excluding the study medication [52]; (f) inability to use the mobile phone application; (g) pregnant or breastfeeding women. 

## 3. Procedures

### 3.1. Study Procedure

**Figure 1:**
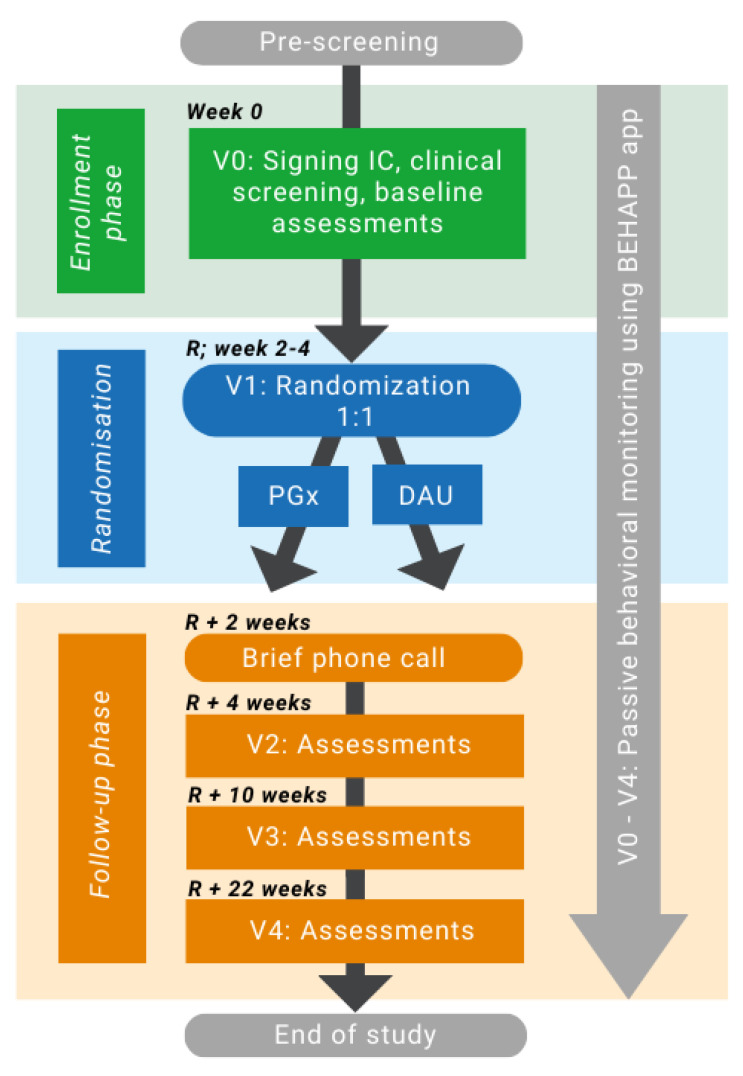
Study flow chart. The study period comprises 24–26 weeks and includes a baseline visit, a randomization visit, three follow-up visits, and a phone call. Behavioral monitoring using the Behapp application [54] will be conducted during the whole study period. Baseline visit (V0) and Visit two assessments include somatic measurements (ECG, blood sampling, weight, blood pressure, heart rate), clinical modulators, and questionnaires. Visit 3 (V3) and 4 (V4) assessments include clinical modulators, anthropometric measurements (weight, blood pressure, heart rate), and psychometric questionnaires. Abbreviations: IC = informed consent; DAU = dosing as a usual group; PGx = pharmacogenetic-guided group; R = randomization; V = visit.

The study comprises a baseline visit, a randomization visit, three follow-up visits, and one phone call. The study flow is depicted in Figure 1 (for details, see Table S1 in Supplementary Materials). Patients will be approached by the treating physician at the local inclusion sites. At the baseline visit (V0), all participants will be asked to sign an informed consent form prior to inclusion, which will be co-signed by the investigator. After inclusion, baseline assessments will be performed on the same day, and blood samples will be taken to determine the pharmacogenetic profile and therapeutic drug monitoring analyses. Patients will be randomized (V1) between two to four weeks later. V1 can be a visit or also a short phone call, in which participants will be randomized to a “treatment guided by pharmacogenetic testing” (PGx) group or to a “dosing as usual” (DAU) group with a 1:1 ratio for allocation to either group. Randomization will be stratified by diagnosis (mood, anxiety, and psychotic disorder) and carried out using the Electronic Case Report Form (eCRF) Castor EDC [53]. Participants and raters will be blinded throughout the study. Medication will be prescribed by the participants’ prescribing clinician, either a psychiatrist or a general physician. Until randomization, patients will continue to use their current medication. If necessary, medication can be started in the lowest dose already before randomization. From randomization onwards, prescribing physicians in both treatment groups may alter a patient’s psychotropic medication regimen in terms of the type of medication, dose, schedule, or number of medications if necessary. The medication and dosages used will be recorded at each visit.

To compare pharmacotherapy prescribed as usual with personalized treatment using pharmacogenetic information, all enrolled patients will be followed up after baseline (V0) for a 24-week period and will be assessed at randomization (V1), 4 weeks post-randomization (V2), 10 weeks post-randomization (V3), 22 weeks post-randomization (V4; Table S1 in Supplementary Materials). Two weeks after randomization, a telephone call will be made with the participant to increase motivation to maintain medication adherence and answer potential questions. During V2, V3, and V4, follow-up assessments will be conducted, including self-reported measures of medication efficacy. The estimated window for the follow-up visits is ±2 days for V2, ±5 days for V3, and ±7 days for V4, always in relation to V1, during which randomization takes place. All study procedures and assessments will be conducted by trained investigators. After the end of the study, participants in both the PGx and DAU groups will be informed about their pharmacogenetic profile. 

### 3.2. Study Readouts (Table S1 in Supplementary Materials)

#### 3.2.1. Psychiatric Evaluation

Psychiatric diagnoses will be assessed using MINI (Mini international neuropsychiatric interview) version 7.0.2 for DSM-5 [51].

#### 3.2.2. Patient Characteristics

The following patient characteristics will be assessed: age, sex, demographics (including ethnicity, living conditions, marital status, education, occupation, parental education, parental occupation), medication history (including duration, dosages, inefficacy, and side effects), family history of psychiatric illness (including medications, inefficacy, and side effects), diet, smoking, alcohol use, and comorbid conditions. Additional clinical modulators will be evaluated, including duration of illness episode, baseline symptom severity, suicidal ideation, and level of anxiety, since these have been described to influence treatment response [55,56,57]. 

#### 3.2.3. Primary and Secondary Outcomes

The primary outcome is personal recovery as assessed by the patient at 24 weeks, using the recovery assessment scale (RAS domains and stages), which is a self-reported questionnaire [58,59]. Over the last few years, there has been a shift towards patient-oriented recovery, such as personal recovery, which focuses more on living a meaningful life despite the potential constraints of one’s psychiatric disorder rather than clinical improvement. The RAS-DS appears to have solid psychometric and conceptual features that make it useful in psychiatric health services research [60]. 

Secondary outcomes that are examined will be measured over a 24 (up to 26) week period (V0, V2, V3, and V4) and will include clinical effects (response or symptomatic remission), side effects, general well-being, and psychosocial functioning. Clinical symptomatology will be evaluated using the Structured Interview Guide for the Hamilton Depression Rating Scale (SIGH-D) for participants with mood disorders [61], the Structured Interview Guide for the Hamilton Anxiety Rating Scale (SIGH-A) for anxiety disorders [62], and the Positive and Negative Syndrome Scale (PANSS) for psychotic disorders [63]. The response will be defined as a 50%-point reduction in the SIGH-D for depressive disorders, SIGH-A for anxiety disorders, or PANSS for psychotic disorders. Symptomatic remission is defined as a SIGH-D score of 7 or less, a SIGH-A score of 7 or less, and a PANSS score of 57 or less. Side effects will be assessed using both the Frequency, Intensity, and Burden of Side Effects Ratings (FIBSER) [64] and the *Udvalg for Kliniske Undersogelse*—Side Effects Rating Scale (UKU-SERS) [65]. General well-being and quality of life will be assessed by means of the EuroQol 5 Dimensions-5 levels questionnaire (EQ-5D-5L) [66] and psychosocial functioning by means of the Functioning Assessment Short Test (FAST) [67]. Current medication use will be recorded at each visit (V0, V1, V2, V3, V4) and include information on type, dose, target symptoms, side effects, and effectivity.

#### 3.2.4. Anthropometric Readouts

Blood samples will be collected by taking blood from the participants according to a standard medical procedure (venipuncture) for clinical chemistry and therapeutic drug monitoring (TDM) at V0 and V2 and for genotyping purposes at baseline (V0). An ECG will be performed at V0 and V2. Other somatic and medical measures include hip-waist circumference, height, weight, blood pressure, and blood pulse, which will be collected at weeks 0, 6, 12, and 24 (V0, V2, V3, and V4, respectively). 

#### 3.2.5. Clinical Chemistry and Therapeutic Drug Monitoring (TDM)

Blood samples at V0 and V2 will be analyzed by performing standard laboratory blood tests. The blood will be processed by centrifuging and supernatant collection immediately after collection to obtain serum for medical biochemistry tests and plasma for the TDM. The biochemistry assessment will include levels of glucose, sodium, creatinine, ALAT/ASAT, GGT, fatty lipids, prolactin, TSH, Hb/MCV, vitamin B12 and D, and folic acid. Additionally, at the end of the study, all blood samples collected at V0 and V2 will be used for TDM at the research site in Belgrade, Serbia (FFUB). Steady-state blood concentrations of the prescribed medications (sertraline, escitalopram, risperidone, or aripiprazole) in blood plasma will be measured using high-performance liquid chromatography with tandem mass spectroscopy (HPLC-MS/MS) according to the previously validated TDM protocol for respective drugs [68,69].

#### 3.2.6. Passive Behavioral Monitoring

The Behapp mobile application will be used to perform passive behavioral monitoring of participants [54]. As monitoring behavioral data can assist in predicting relapse and guide future interventions in preventing relapse [69], the Behapp data will be used for more extensive phenotyping of the participants to contribute to further personalizing the pharmacotherapy approach in psychiatry. Passive monitoring using Behapp has been validated for research in psychiatric patients [70] (Table S2 in Supplementary Materials). This app is currently not used in standard clinical practice. 

### 3.3. Genotyping

After blood collection, the samples will be pseudonymized and transported from the clinical sites to a central genotyping facility in Bonn. Genotyping will be performed using either Polymerase Chain Reaction (custom TAQMan open array panel, ThermoFisher, Waltham, MA, USA) or a genome-wide microarray analysis including specific pharmacogenetic content (Infinium Global Diversity Array with Enhanced PGx; Illumina, San Diego, CA, USA). Both technologies contain detailed information on genotypes relevant to pharmacogenetics. The genotypes tested will include all relevant common and some rare functional allelic variants, including those that are expected to be prevalent within our study population [50]. For CYP2C19, these include alleles implicated in absent enzymic function (*CYP2C19Null; alleles* *2, *3, *4, *5, *6, *8), as well as increased function (*CYP2C19Inc*; allele *17). Regarding expression of CYP2D6, absent enzymic function (*CYP2D6Null*; alleles *3, *4, *5, *6, *7, *8, *11, *12, *14, *18, *19, *20, *29, *38, and *42), reduced function (*CYP2D6Red*; *9, *10, *17, and *41), and increased function (through CYP2D6 multiplication; *CYP2D6xN*) are included (Table 1). Interpretations and validation of the genotyping results are performed continuously in the central laboratory. 

### 3.4. Dosing Recommendations 

After genotyping, the prescribing clinician will receive dosing recommendations (in case the patient is randomized into the PGx group) at V1 within two to four weeks after the baseline visit (V0). Antidepressants escitalopram or sertraline will be prescribed for mood- and anxiety disorders, and antipsychotics risperidone or aripiprazole for psychotic disorders, as these drugs are commonly used in all countries participating and sufficient scientific evidence for genotype-based dosage recommendations is present [71]. The categorization and dosing recommendations are based on recently published results from over 5000 patients (Table 2) [43,71,72] and according to guidelines of the Dutch Pharmacogenetic Working Group (DPWG) [14,40,41] and the Clinical Pharmacogenetics Implementation Consortium (CPIC) [15,16].

### 3.5. Sample Size and Power Calculation 

The current study aims to recruit a total of 2500 participants, including 1250 patients with mood disorders, 750 patients with anxiety disorders, and 500 patients with psychotic disorders. Based on previous prospective pharmacogenetic studies in psychiatry [32], a drop-out rate of 10% after screening and an additional drop-out rate of 20% after randomization is estimated over a period of 2 years. The number of participants to be recruited per diagnosis is divided per center based on their estimation of patients’ willingness to participate in clinical studies and the estimated number of patients that each research center indicated to be able to recruit. This sample size allows us to detect a small effect of 0.15 (Cohen’s *d*), which could be detected in a trans-diagnostic independent group’s comparison (*t*-test) with a power of 90%. Assessment of the outcomes within each diagnostic group, with adjustment of the alpha level for multiple comparisons, gives 90% power to detect a between-groups difference of 0.23 (small) for mood disorders, 0.3 (small to moderate) for anxiety disorders, and 0.36 (moderate) for psychotic disorders.

### 3.6. Statistical Analysis 

A descriptive analysis will summarize patient characteristics at baseline, also including primary and secondary outcomes. The data analyst, an independent statistician, will be blinded to patient allocation and will not participate in data collection. 

The statistical analyses will be carried out using linear mixed-effects models according to the intention to treat (ITT) principle. The model will be adjusted for several important covariates (i.e., sex, age, ethnicity) that are plausibly predictive of missingness. The model should be robust for complete missingness at random. The model corrects for this and will yield valid results if missingness can be predicted depending on the diagnostic group to which one belongs. Reasons for drop-out will be documented. The analyses of the primary outcome, assessing patient recovery as measured using the RAS-DS, will be carried out using a mixed effects model with a main effect for group and time (continuously), including the interaction effect of group x time. The model will include a random intercept for participants and a random slope for time. The secondary outcomes will be analyzed using a three-way interaction with group, time, and diagnosis to assess differences in time trends across diagnoses. If significant, post hoc analyses with pairwise contrasts will be conducted to identify in which diagnosis group a difference in time trend is found. All analyses will be performed separately for each diagnostic group.

An interim analysis will be performed after 50% of participants are included. In case our primary research objective can be answered, the study will be prematurely terminated. The Data Safety Monitoring Board (DSMB) will evaluate the interim data and has a further advising role. 

### 3.7. Overarching Project 

The clinical study described in the study protocol is part of the PSY-PGx project (www.PSY-PGx.org, accessed on 17 January 2024). This overarching project aims to combine the outcomes of the current work with existing pharmacogenetic biobank data to generate a medication prescription algorithm based on machine learning principles. A first medication algorithm will be based solely on existing biobank data of the UK Biobank [73] and the seven Finnish Biobanks [74], which will be further developed using the de-identified data from the current study. Moreover, the biobanks will be assessed to identify potential pharmacogenes and proxies for clinical outcomes. Besides pharmacogenetic factors, additional individual patient characteristics that mediate individual medication responses will be evaluated and included in the medication algorithm. Finally, the PSY-PGx consortium aims to set up a DNA biobank to allow for future pharmacogenetic research. The DNA biobank will be established in collaboration with Systasy Bioscience GmbH (Munich, Germany), which developed an extensive cellular biobank infrastructure comprising biological samples from patients with psychiatric disorders. An additional optional written consent is obtained from participants in order to use their data for these purposes. The PSY-PGx project is coordinated by chief principal investigator RvW at Parnassia Psychiatric Institute, comprises 16 global partners, and is funded by the European Union’s Horizon 2020 research and innovation program (grant agreement No 945151). 

## 4. Expected Results

This is the first non-industry-sponsored, large-scale international study to investigate the clinical utility of pharmacogenetic-guided treatment in patients with psychotic disorders, anxiety disorders, and depressive disorders in the frame of precision psychiatry [75,76]. There is an urgent need for a more effective and personalized approach to the treatment of psychiatric disorders. Although an increasing number of studies have indicated a positive relation between the use of pharmacogenetics and improved clinical outcomes in psychiatry [26,27,28,29,30,31,32,33,43] and dosing recommendations are available [14,15,16], the implementation of these guidelines in clinical practice, in general, is still lacking due to limited evidence of improved clinical outcome in randomized clinical trials. To that end, the current study aims to provide further evidence of the clinical utility of pharmacogenetic testing in psychiatry. Additionally, this work will also contribute to our understanding of pharmaco-genes and other potential phenotypic biomarkers that are involved in the effect of (medication) therapies. As previous studies have mostly focused on the use of pharmacogenetics in antidepressants in patients with mood disorders, this study will provide insights into the clinical use of pharmacogenetics in the treatment of mood-, psychotic, and anxiety disorders. To our knowledge, this is the first prospective, randomized, patient- and rater-blinded, and non-industry-sponsored study investigating pharmacogenetic-guided treatment dosing for psychiatric disorders. 

We hypothesize that patients in the pharmacogenetic-guided treatment group (PGx group) will be treated more effectively and will experience fewer side effects than patients who will be treated according to dosing as usual recommendations (DAU group). Our primary outcome entails patient-measured recovery assessed using the RAS-DS, which use has been shown to allow for a more personal recovery-oriented method of measuring clinical outcomes [58]. We will investigate if this finding is also reflected in better patient recovery as measured by the participants themselves. As this study has a longer follow-up than previous pharmacogenetic studies (24 weeks in this study, compared to 12 weeks in most others), we expect to detect a larger effect than previous studies reported. Additionally, we expect to find this effect more clearly in this study, as we include participants with moderate to severe disease severity who have a history of inadequate response or intolerability to at least one type of pharmacotherapy, a population that is expected to benefit most from pharmacogenetic testing.

In regard to our secondary objectives, we hypothesize that participants in the PGx group will experience greater symptom improvement, larger response and remission rates, and fewer side effects compared to the DAU group. In addition, we expect these findings to be reflected in better general well-being and psychosocial functioning. Finally, we expect that the results of this study will further identify phenotypical characteristics that may be involved in the prediction of clinical outcomes. For most psychiatric disorders, individual treatment needs are not well defined, and therefore, it seems essential to conduct studies that include a broader range of information regarding personalized treatment besides pharmacogenetics. We expect to identify and determine the extent to which other factors may influence medication response, including (in)efficacy or the emergence of adverse effects, such as sex, age, comorbidity, co-medication, and specific behavior as assessed via passive behavioral monitoring (via the Behapp-app), such as the use of mobile phone or action radius. By more extensively phenotyping patients, we will contribute to further developing a personalized pharmacotherapy approach toward better and safer treatment outcomes in psychiatry.

## 5. Strengths and Limitations

The strengths of the present study include the prospective randomized controlled patient and rater-blinded design, which allow us to determine the association between pharmacogenetic-guided treatment and clinical outcome with greater power. In contrast to prior prospective studies investigating pharmacogenetics in psychiatry, this study is not industry-sponsored, decreasing the risk of bias, as compared to more favorable outcomes that are potentially associated with previous industry-sponsored studies [77]. In contrast to previous pharmacogenetic trials, we here blind both the participants and raters for the pharmacogenetic phenotype. Another strength of the current study is that the information generated in this study will be used to strengthen an algorithm for the personalized treatment of psychiatric patients further. The phenotypical characteristics, as well as pharmacogenetic factors that are found to mediate medication response, will be used to enrich a medication prescription algorithm based on machine learning principles, which will be set up by the PSY-PGx consortium using existing biobank data. Finally, patients with polypharmacy (>5 medications) were excluded, which allows us to better investigate the efficacy and adverse events of the antidepressants and antipsychotics studied here.

A limitation is that we will not extensively evaluate the symptomatic severity of secondary diagnoses by adding additional questionnaires in the case of comorbid patients; however, we evaluate double diagnoses at baseline. A second limitation is that therapy choices are restricted to only two treatment options per diagnosis, limiting results and disregarding other pharmacotherapeutics that have been found to be metabolized by the cytochrome P450 enzymes. Another limitation is that only the genotype status of *CYP2C19* and *CYP2D6* are evaluated for the treatment decision. However, the allelic variants of these genotypes are relevant for the medications studied in this trial [6,14], and only for these two is there solid scientific evidence that it influences clinical outcomes in psychiatric patients, and we, therefore, do not expect this to affect our primary outcome. Future studies should broaden the treatment options and evaluate more clinically relevant genotypes. Results from this study may provide further insights into which genes may be implicated in medication response, and these could be included in future studies. 

## 6. Ethics and Dissemination

The results of this study will be published in peer-reviewed scientific journals that have open-access options. The current work will also be disseminated at national and international conferences. The PSY-PGx consortium has set up a work package dedicated to Dissemination and Exploitation, working with the European patient advisory organization GAMIAN-Europe (Global Alliance of Mental Illness Advocacy Networks-Europe, Brussels, Belgium).

All procedures contributing to this work comply with the ethical standards of the relevant national and institutional committees on human experimentation and are in line with the Helsinki Declaration of 1975 (revised in 2008). The coordinating site has received approval from the Medical Ethics Committee at Maastricht University (reference number NL79649.068.21/METC21-081). All procedures have been or will be approved by local ethics committees in each of the participating countries prior to any study-related activities, using either local ethics procedures or via CTIS. The study was registered at ClinicalTrials.gov (accessed on 17 January 2024, Identifier: NCT05656469). Written informed consent is obtained from each participant before any study-related activities will be conducted. 

## Figures and Tables

**Table 1:** *CYP2D6* and *CYP2C19* gene variants.

Genotype	Enzyme Function	Alleles
*CYP2C19Null*	Loss of function	CYP2C19*2, *3, *4, *5, *6, *8
*CYP2C19Inc*	Increase	CYP2C19*17
*CYP2D6Null*	Loss of function	CYP2D6*3, *4, *5, *6, *7, *8, *11, *12, *14, *18, *19, *20, *29, *38, *42
*CYP2D6Red*	Reduction	CYP2D6 *9, *10, *17, and *41
*CYP2D6xN*	Increase	*CYP2D6* multiplication

**Table 2:** Predefined dosing schedule per genotype (based on data from [43,72,73]).

CYP2C19	Genotype	Escitalopram *Daily Dose*	Sertraline *Daily Dose*	CYP2D6	Genotype	Risperidone *Daily Dose*	Aripiprazole *Daily Dose*
Poor (PM)	Null/Null	5 mg	50 mg	Poor (PM)	Null/Null	3–4 mg 4 mg	15–20 mg >10 mg Max 10 mg
Intermediate (IM)	Wt/Null Inc/Null	5–10 mg	100 mg	Intermediate (IM)	Null/Red Red/Red	4–5 mg	20–25 mg
				Intermediate (IM+)	Wt/Null	5–6 mg	25–30 mg
Normal (NM)	Wt/Wt	15–20 mg	100–150 mg	Normal (NM)	Wt/Wt	6 mg	30 mg
Ultrarapid (UM)	Wt/Inc Inc/Inc	15–20 mg	100–150 mg	Ultrarapid (UM)	Wt/Wt × 2	6 mg Avoid or 12 mg	30 mg

## Supplementary Materials

The following supporting information can be downloaded at: https://www.mdpi.com/article/10.3390/ph17020151/s1, Table S1: Visit schedule; Table S2. Behapp is an integral part of the new PSY-PGx model of care.
