# Supplementary material for: A New Intervention for Implementation of Pharmacogenetics in Psychiatry: A Description of the PSY-PGx Clinical Study
Source: Pharmaceuticals (Basel). 2024 Jan 23;17(2):151. doi: 10.3390/ph17020151 (PMC10892863; doi:10.3390/ph17020151)
Supplement: Supplementary file 1 [file pharmaceuticals-17-00151-s001.zip › pharmaceuticals-2782010-supplementary.pdf]

Supplemental Table S1

Table S1: Visit schedule

**Assessments**	**V0**	**V1***	**V2 (week 6)****	**V3 (week 12)*****	**V4 (week 24)******
**Duration of visit**	2 hours	10 min	1 hour	1 hour	1.5 hour
**Psychiatric assessment and MINI**	+				
**Patient Characteristics**	Age, gender, demographics, diet, smoking, drugs of abuse, alcohol, comorbidity, family history		Smoking, drugs, alcohol		Smoking, drugs, alcohol
**Medication**	Medication history (including inefficacy and side effects), treatment duration, dosages, current medication, family history of medication	Check current medication	Check current medication	Check current medication	Check current medication
**Clinical Modulators**	Long duration of illness episode, higher baseline severity, moderate to severe suicidal ideation, high level of anxiety	Re-assess clinical modulators such as suicidal ideation and anxiety levels	Re-assess clinical modulators such as suicidal ideation and anxiety levels	Re-assess clinical modulators such as suicidal ideation and anxiety levels	Re-assess clinical modulators such as suicidal ideation and anxiety levels
**Somatic Measurements**	Hip-waist circumference, height, weight, blood pressure, pulse, ECG		Hip-waist circumference, weight, blood pressure, pulse, ECG	Hip-waist circumference, weight, blood pressure, pulse	Hip-waist circumference, weight, blood pressure, pulse
**Blood measurements**	Genotyping, clinical chemistry, Therapeutic drug monitoring (TDM)		Clinical chemistry, Therapeutic drug monitoring (TDM)		

**Questionnaires**	SIGH-D, SIGH-A or PANSS (depending on diagnosis), FIBSER, UKU, EQ-5D-5L, FAST, RAS-DS		SIGH-D, SIGH-A or PANSS (depending on diagnosis), FIBSER, UKU, EQ-5D-5L, FAST, RAS-DS	SIGH-D, SIGH-A or PANSS (depending on diagnosis), FIBSER, UKU, EQ-5D-5L, FAST, RAS-DS	SIGH-D, SIGH-A or PANSS (depending on diagnosis), FIBSER, UKU, EQ-5D-5L, FAST, RAS-DS
**IT data**	**Behapp app for passive monitoring**: throughout the duration of the trial period.				

Note *max. four weeks after baseline. The estimated window for the follow up visits and the telephone call is ± **2 days, *** ± 5 days, **** ± 7 days, always in relation to visit V1 during which randomisation takes place.

randomisation visit (V1). Two weeks after V1, a brief phone call will be carried out to evaluate initial response to treatment and motivate the patient to continue taking the medication.

Supplemental Table S2

Table S2: Behapp as an integral part of the new PSY-PGx model of care

	BEHAPP
**ICT Solution**	Behapp
**Type**	Mobile App
**Website**	https://www.behapp.com/
**Available languages**	Google provided languages
**Purpose**	Data collection through passive monitoring of patients via app for patients
**Functionalities/ Specifications**	Smartphone application for assessment of typical and abnormal human behavioural phenotypes in their natural environment.
**Innovation**	A passive behavioural monitoring tool from which data has been successfully collected in a wide variety of brain disorders, including depression, schizophrenia, and Alzheimer’s Disease.
**Use in PSY-PGx**	Endpoint measures will be used to develop an implementation strategy of pharmacogenetics-based personalised medicine in psychiatry at an international level.

**Technology Readiness Level (TRL)**	Currently: TRL 7 [57]<br><br>Proof of concept data has been collected and analysed as part of IMI PRISM project.<br><br>Initiated path forward to informally discuss a digital biomarker using this technology with the EMA innovative task force (ITF)
**Previous tests**	App has been applied in a variety of disease cohorts (Schizophrenia, Depression, and Alzheimer's disease) in a variety of national and EU funded projects (e.g., IMI PRISM, IMI ROADMAP, SMARD).
